# Modeling specific action potentials in the human atria based on a minimal single-cell model

**DOI:** 10.1371/journal.pone.0190448

**Published:** 2018-01-23

**Authors:** Yvonne Richter, Pedro G. Lind, Philipp Maass

**Affiliations:** Fachbereich Physik, Universität Osnabrück, Barbarastraße 7, 49076 Osnabrück, Germany; University of Minnesota, UNITED STATES

## Abstract

We present an effective method to model empirical action potentials of specific patients in the human atria based on the minimal model of Bueno-Orovio, Cherry and Fenton adapted to atrial electrophysiology. In this model, three ionic are currents introduced, where each of it is governed by a characteristic time scale. By applying a nonlinear optimization procedure, a best combination of the respective time scales is determined, which allows one to reproduce specific action potentials with a given amplitude, width and shape. Possible applications for supporting clinical diagnosis are pointed out.

## Introduction

Detailed reaction-diffusion models to describe human atrial electrophysiology were first developed in the late 1990s [1–4] and are further developed until now. Important steps forward have been made to include specific ionic currents [5–10], which in particular allow one to investigate specific effects of pharmaceuticals in treatments of atrial fibrillation and other heart failures. Complementary to these detailed models, Bueno-Orovio, Cherry and Fenton introduced in 2008 a minimal reaction-diffusion model (BOCF model) for action potentials (AP) in ventricular electrophysiology, where the large number of ionic currents through cell membranes is reduced to three net currents [11]. This model has four state variables, one describing the transmembrane voltage (TMV), and the other three describing the gating of ionic currents. The TMV, as in detailed reaction models, satisfies a partial differential equation of diffusion type with the currents acting as source terms, and the time evolution of the gating variables is described by three ordinary differential equations coupled to the TMV. By fitting the action potential duration (APD), the effective refractory period and the conduction velocity to the detailed model of Courtemanche, Ramirez and Nattel [1] (CRN model), the BOCF model was recently adapted to atrial electrophysiology (BOCF model) [12].

**Fig 1:**
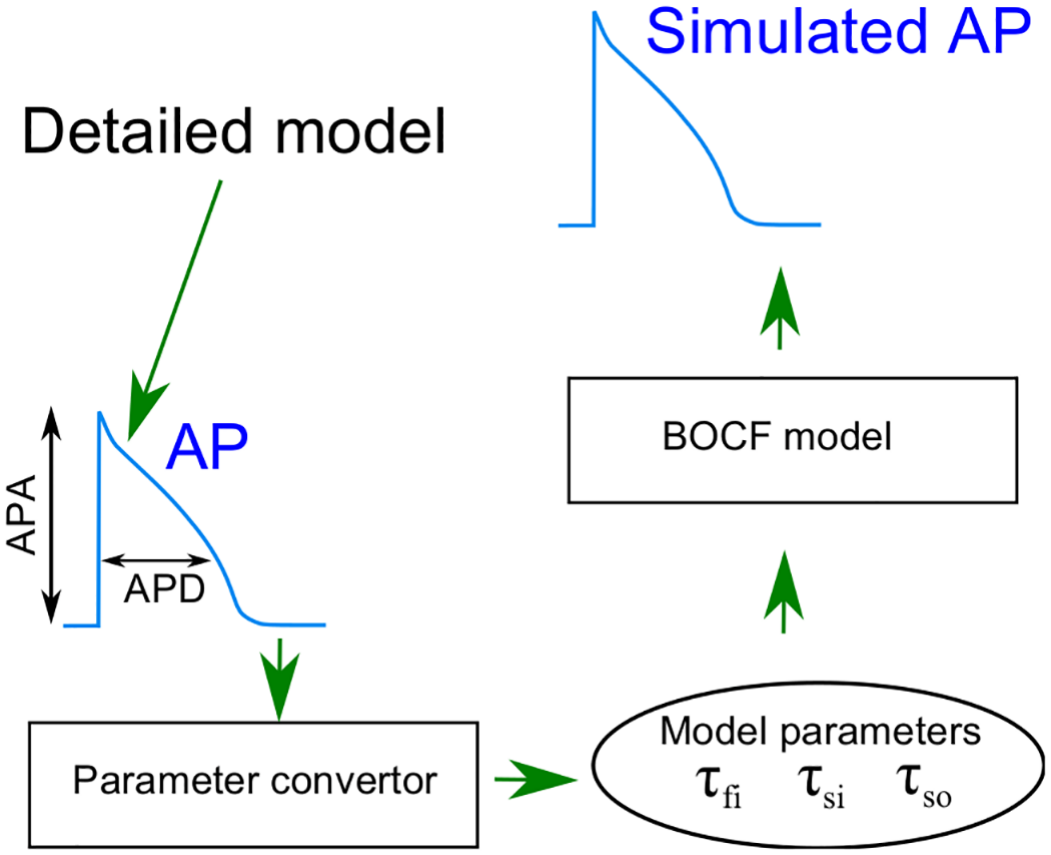
Overview. Schematic illustration of the optimized adjustment of the BOCF model by a parameter converter that determines the set of parameter values (*τ*_fi_, *τ*_si_, and *τ*_so1_) giving a best match with the amplitude and duration of a specific action potential.

**Fig 2:**
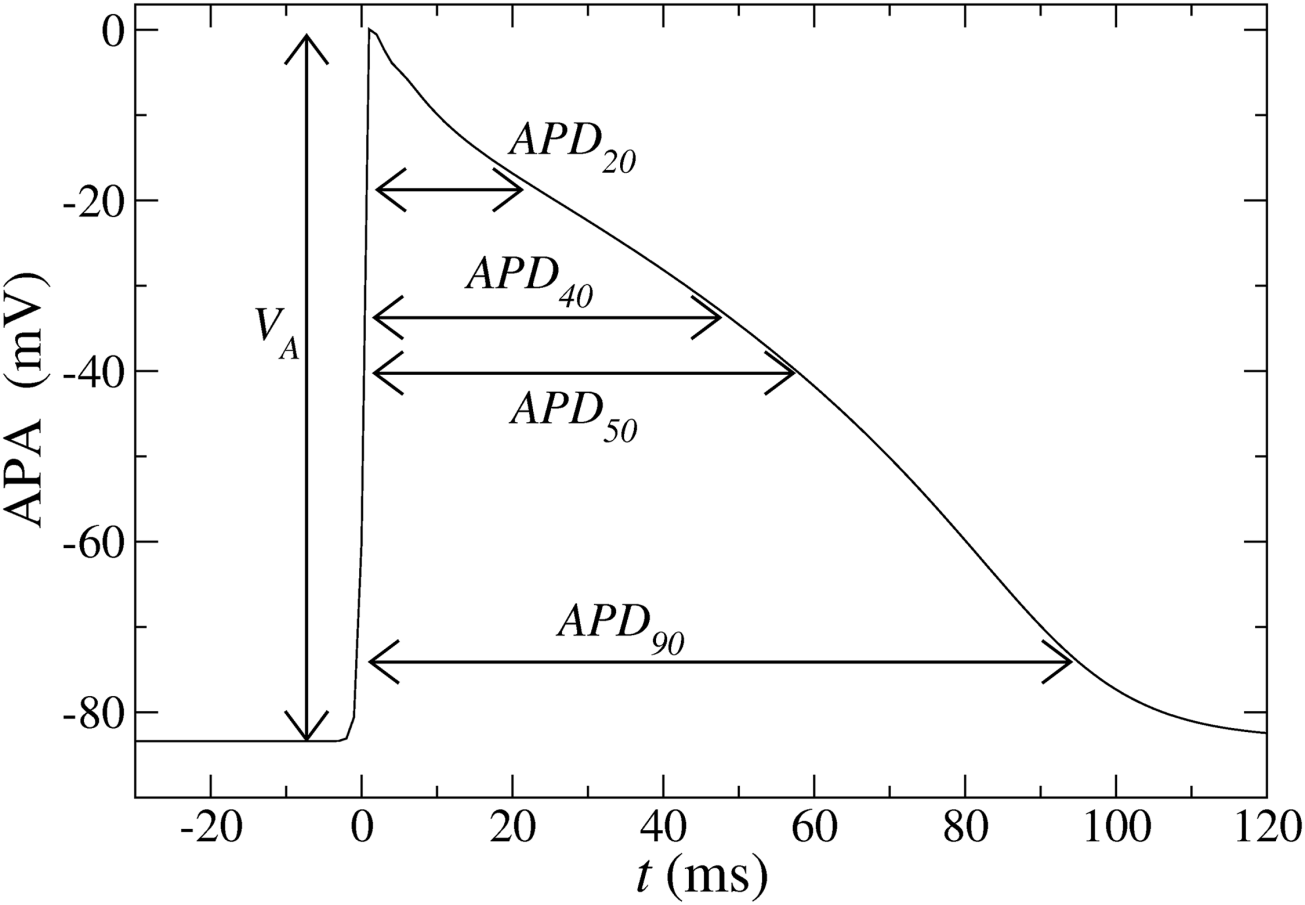
Illustration of an action potential. The amplitude APA and four AP durations at 90%, 50%, 40% and 20% of the total amplitude are also indicated. These five values are used to determine three characteristic time scales of the BOCF model (see text).

In this work we develop a method to model specific AP based on the BOCF model as it is aimed in the clinical context in connection with improved and extended possibilities of diagnosis [13]. Compared to the detailed models, the BOCF model has the advantage that it is better amenable to some analytical treatment. This allows us to identify a small set of relevant model parameters for capturing the main features of a specific AP. Our methodology is sketched in Fig 1 and can be summarized as follows. We start by labeling each given AP with its amplitude APA and with four APD, namely at 90%, 50%, 40% and 20% repolarization, denoted as APD_90_, APD_50_, APD_40_, and APD_20_ respectively. These APD_*n*_ (*n* = 20, 40, 50, 90) together with the amplitude APA are suitable to catch a typical shape of a specific AP, see Fig 2.

The APD_*n*_ taken for a specific patient are given to a parameter convertor that retrieves specific parameter values of the BOCF model. As relevant parameters, we adjust three time scales governing the closing and opening of the ionic channels. The parameter convertor consists of an optimization algorithm that searches for the best set of parameter values consistent with the measured AP properties.

The paper is organized as follows. In Section “BOCF model for atrial physiology” we shortly summarize the BOCF model and discuss the role of the three fit parameters that we selected to model specific AP. In Section “Parameter dependence of BOCF action potentials” we show how these parameters can be adjusted to obtain a a faithful representation of the AP properties APA, APD_*n*_, and in Section “Modeling of patient-specific action potentials with the BOCF model” we demonstrate the specific AP modeling for surrogate data generated with the CRN model [1]. A summary of our main findings and discussion of their relevance is given in Section “Conclusions”. In the supporting information, we provide analytical calculations for the BOCF model that motivated our choice of fit parameters for the AP modeling. We also analyze the robustness of the optimization procedure with the activation frequency.

## BOCF model for atrial physiology

The BOCF model has four state variables, which are the scaled TMV *u*, and three variables *v*, *w* and *s* describing the gating of (effective) net currents through the cell membrane. The TMV *V* is obtained from *u* via the linear relation *V* = *V*_*R*_(1 + *αu*), where for atrial tissue we set *V*_*R*_ = −84.1 mV for the resting potential and *α* = 1.02 [12]. The time-evolution of *u* is given by the single-cell action potential model, here defined as

$$\begin{array}{c} \partial_{t}u=J\left(u,v,w,s\right)+J_{\text{stim}}\;, \end{array}$$ (1)

where *J* = *J*(*u*, *v*, *w*, *s*) is the total ionic current and *J*_stim_ an external stimulus current. The total ionic current decomposes into three net currents, a fast inward sodium current *J*_fi_ = *J*_fi_(*u*, *v*), a slow inward calcium current *J*_si_(*u*, *w*, *s*), and a slow outward potassium current *J*_so_ = *J*_so_(*u*),

$$\begin{array}{c} J\left(u,v,w,s\right)=J_{\text{fi}}\left(u,v\right)+J_{\text{si}}\left(u,w,s\right)+J_{\text{so}}\left(u\right)\;. \end{array}$$ (2)

These currents are controlled by the gating variables, which evolve according to

$$\begin{array}{c} \partial_{t}\left(v,w,s\right)=\left(E\left(u,v\right),F\left(u,w\right),G\left(u,s\right)\right)\;, \end{array}$$ (3)

where the nonlinear functions *F*, *G* and *H*, are specified in S1 Appendix. There we show that the four differential Eqs (1) and (3) can be reduced to a system of two differential equations. This reduction shows that the three characteristic times *τ*_fi_, *τ*_si_ and *τ*_so1_, which fix the typical duration of the respective currents, govern the shape of the AP [cf. S1 Appendix]. We take these three time scales as parameters for fitting a specific AP and keep all other parameters fixed. For the values of the fixed parameters we here consider the set determined for the electrically remodeled tissue due to atrial fibrillation [12, 14].

## Parameter dependence of BOCF action potentials

In this section we show that in the BOCF model the amplitude APA can be expressed by a quadratic polynomial of the times *τ*_fi_, and the APD_*n*_ by cubic polynomials of *τ*_si_ and *τ*_so1_.

The dependence of APA and the APD_*n*_ on the characteristic times, was determined from generated AP in single-cell simulations of the BOCF model by applying periodically, with a frequency *f* = 3 Hz, a square stimulus current of 40 pA, corresponding to an amplitude of 4.76 s^−1^ for the current *J*_stim_ in Eq (1), for a time period of 3.5 ms. The resulting time evolution of the TMV in response to this stimulus was calculated by integrating Eqs (1) and (3) for the initial conditions *u*_0_ = 0, *v*_0_ = 1, *w*_0_ = 1 and *s*_0_ = 0. This was done for (*τ*_fi_, *τ*_si_, *τ*_so1_)∈[0.002, 0.210] × [5.9, 22.4] × [40, 110] (in ms) with a resolution Δ*τ*_fi_ = 0.0021 ms (100 values), Δ*τ*_si_ = 0.3 ms (56 values) and Δ*τ*_so1_ = 1 ms (71 values). The AP was recorded after a transient time of 10 s.

**Fig 3:**
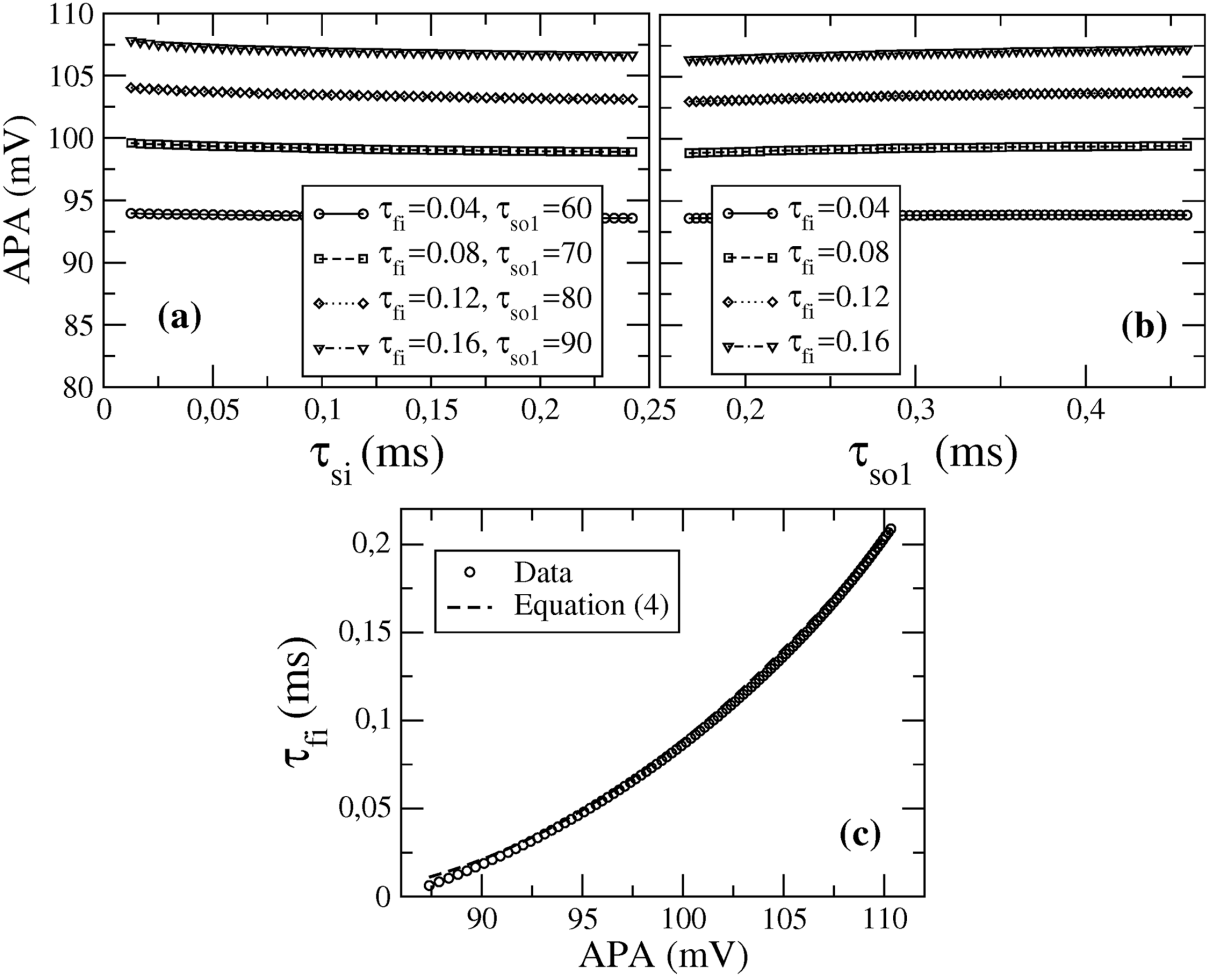
Amplitude as function of model parameters. (a) Amplitude APA as a function of *τ*_si_ for four different pairs of fixed values *τ*_fi_ and *τ*_so1_ (in ms). (b) Dependence of the amplitude APA on time *τ*_so1_ for *τ*_si_ = 10.7 ms and four different values of *τ*_fi_. (c) Time *τ*_fi_ as a function of APA for *τ*_si_ = 10.7 ms and *τ*_so1_ = 73.7 ms.

As shown for a few representative pairs of fixed values of *τ*_fi_ and *τ*_so1_ in Fig 3(a) and 3(b), the APA depends only very weakly on *τ*_si_ and *τ*_so1_. Neglecting these weak dependencies, on *τ*_si_ and *τ*_so1_, we find the APA to increase monotonically with *τ*_fi_ in the range [85, 110] mV relevant for human atria. In Fig 3(c) we show that the parameter *τ*_fi_ can be well described by the quadratic polynomial

$$\begin{array}{c} \tau_{\text{fi}}=c_{0}{\text{APA}}^{2}+c_{1}\text{APA}+c_{2}\;, \end{array}$$ (4)

where the coefficients *c*_*i*_ and the coefficient of determination *R*^2^ of the fit are given in Table 1.

**Table 1:** Polynomial coefficients and *R*^2^ values of the fits of APA to Eq (4) and of the surfaces APD_*n*(*τ*_si_, *τ*_so1_)_ to Eq (5). The values of coefficients $c_{mk}^{\left(n\right)}$ are given in units of mV/(ms)^*m*+*k*^.

Coeffs. Eq (4)	APA	Coeffs. Eq (5)	APD_90_	APD_50_	APD_40_	APD_20_
*c*_0_ ± Δ*c*_0_	0.000235 ± 6 × 10^−6^	$c_{00}^{\left(n\right)}$ $\pm \Delta c_{00}^{\left(n\right)}$	98 ± 10	85 ± 10	84 ± 10	82 ± 10
*c*_1_ ± Δ*c*_1_	-0.038 ± 0.001	$c_{10}^{\left(n\right)}$ $\pm \Delta c_{10}^{\left(n\right)}$	5.4 ± 0.3	5.0 ± 0.3	4.7 ± 0.4	3.8 ± 0.3
*c*_2_ ± Δ*c*_2_	1.52 ± 0.05	$c_{01}^{\left(n\right)}$ $\pm \Delta c_{01}^{\left(n\right)}$	−33 ± 1	−33 ± 1	−33 ± 1	−32 ± 1
**R**^**2**^	**0.9996**	$c_{20}^{\left(n\right)}$ $\pm \Delta c_{20}^{\left(n\right)}$	0.0001 ± 0.004	−0.0010 ± 0.004	0.0001 ± 0.004	0.003 ± 0.004
		$c_{11}^{\left(n\right)}$ $\pm \Delta c_{11}^{\left(n\right)}$	−0.40 ± 0.01	−0.41 ± 0.01	−0.41 ± 0.01	−0.43 ± 0.01
		$c_{02}^{\left(n\right)}$ $\pm \Delta c_{02}^{\left(n\right)}$	2.47 ± 0.06	2.56 ± 0.06	2.61 ± 0.07	2.85 ± 0.06
		$c_{30}^{\left(n\right)}$ $\pm \Delta c_{30}^{\left(n\right)}$	−0.00007 ± 0.00002	−0.00005 ± 0.00002	−0.00004 ± 0.00002	−0.00002 ± 0.00002
		$c_{21}^{\left(n\right)}$ $\pm \Delta c_{21}^{\left(n\right)}$	0.00125 ± 0.00007	0.00096 ± 0.00007	0.00079 ± 0.00007	0.00018 ± 0.00007
		$c_{12}^{\left(n\right)}$ $\pm \Delta c_{12}^{\left(n\right)}$	0.0027 ± 0.0003	0.0045 ± 0.0003	0.0057 ± 0.0003	0.0103 ± 0.0003
		$c_{03}^{\left(n\right)}$ $\pm \Delta c_{03}^{\left(n\right)}$	−0.045 ± 0.001	−0.050 ± 0.001	−0.053 ± 0.001	−0.069 ± 0.001
		**R**^**2**^	**0.9956**	**0.9938**	**0.9926**	**0.9866**

**Fig 4:**
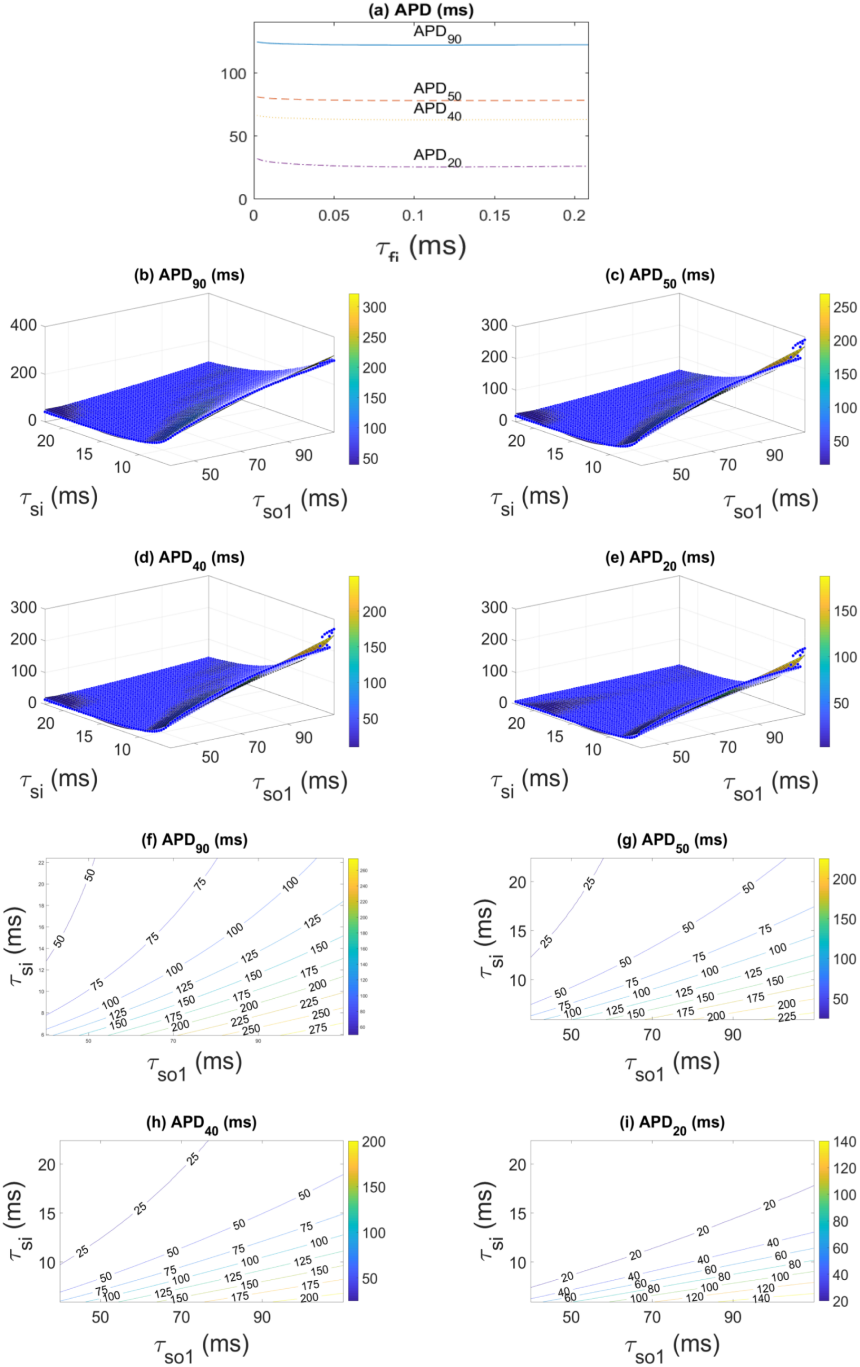
APD_*n*_ as functions of model parameters. (a) APD_*n*_ as a function of *τ*_fi_ for a pair of fixed values *τ*_si_ = 10.7 ms and *τ*_so1_ = 73.675 ms. (b)-(e) Dependence of the APD_*n*_ on *τ*_si_ and *τ*_so1_ for fixed *τ*_fi_ = 0.0835 ms. The meshes of points (black bullets) indicate the simulation results, and the surfaces refer to the fits of the meshes, according to Eq (5). All quantities are given in ms. Plots (f)-(i) show contour plots of the APD surfaces in (b)-(e), respectively.

Likewise, as demonstrated in Fig 4(a) for one fixed pair of values of *τ*_si_ and *τ*_so1_, the APD_*n*_ are almost independent of *τ*_fi_. Their dependence on *τ*_si_ and *τ*_so1_, shown in Fig 4(b)–4(e), can be well fitted by the polynomials

$$\begin{array}{c} {\text{APD}}_{n}\left(\tau_{\text{si}},\tau_{\text{so}1}\right)=\sum_{m=0}^{3}\sum_{k=0}^{3-m}c_{mk}^{\left(n\right)}\tau_{\text{si}}^{m}\tau_{\text{so}1}^{k}\;, \end{array}$$ (5)

where the coefficients $c_{mk}^{\left(n\right)}$ are listed in Table 1 together with the *R*^2^ values of the fits. Fig 4(f)–4(i) display contour plots of the APD surfaces, shown in Fig 4(b)–4(e).

**Fig 5:**
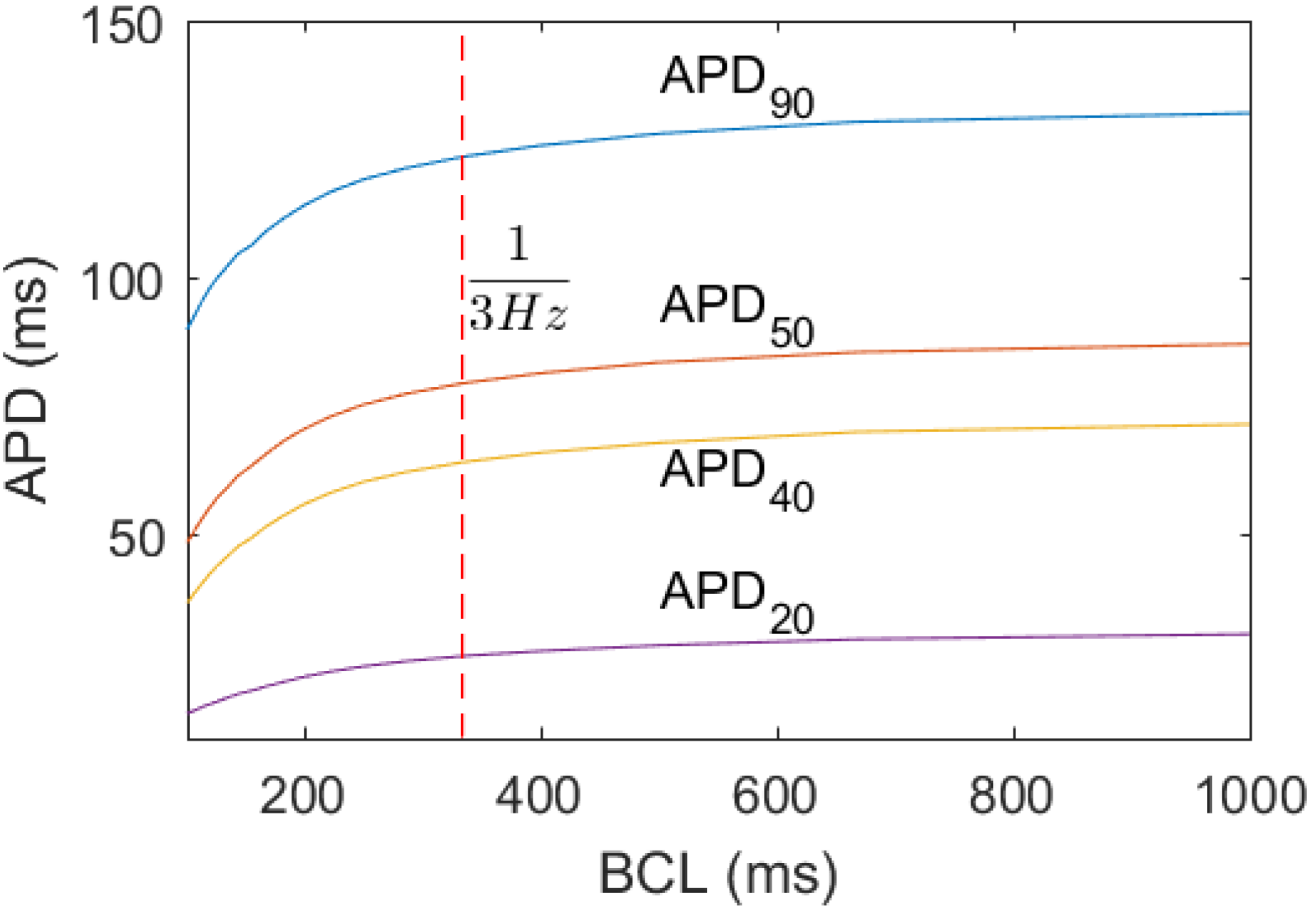
Restitution curves of the BOCF model for APD_90_, APD_50_, APD_40_ and APD_20_, with the remodelled parameter set. The activation frequency of the external stiumulus was varied between 1 Hz and 10 Hz in steps of 1 Hz. The red dashed line indicates the BCL corresponding to *f* = 3 Hz, which we used to illustrate the mapping from APA and APD to the three time scales (see Figs 3 and 4, and Table 1). The AP were taken after 10^4^ ms, beyond the time needed for achieving the stationary state.

The APD_*n*_ of the single cell BOCF model depend on the activation frequency *f* or basic cycle length BCL = 1/*f*. Corresponding restitution curves are shown in Fig 5 for the remodelled tissue. These curves resemble the restitution curves known for others atrial models, see Ref. [15]. With higher frequency (shorter BCL) the APD_*n*_ become smaller. This decrease is more pronounced for frequencies above 6 Hz. As a consequence, the optimization procedure becomes less robust for f≳6 Hz, a feature that is discussed in more detail below in Section “Robustness with respect to the activation frequency”.

## Modeling of patient-specific action potentials with the BOCF model

Let us denote by V the APA and by $D_{n}$ the values of the APD_*n*_ of a specific patient. To model the corresponding AP with the BOCF model, we determine *τ*_fi_ by inserting APA=V in Eq (4) and (*τ*_si_, *τ*_so1_) by minimizing the sum of the squared deviations between the the APD_*n*_, i. e. the function

$$\begin{array}{c} F\left(\tau_{\text{si}},\tau_{\text{so}1}\right)=\sum_{n}{\left[{\text{APD}}_{n}\left(\tau_{\text{si}},\tau_{\text{so}1}\right)-D_{n}\right]}^{2}\;. \end{array}$$ (6)

For the numerical procedure we used the Levenberg-Marquardt algorithm [16]. As one sees from Fig 4(b)–4(e), the APD vary monotonically with the time scales in the ranges fixed above. We checked that the Hessian is positive definite in the corresponding region, implying unique solutions when minimizing F.

To demonstrate the adaptation procedure, we generated surrogate AP with the CRN model [1] for electrically remodeled tissue due to atrial fibrillation [14]. Specifically, we consider the maximal conductances, *g*_Ca_ and *g*_Na_ of the calcium and sodium currents to vary, while keeping all other parameters fixed to the values corresponding to the electrically remodeled tissue. The conductance *g*_Ca_ affects both the AP plateau and the repolarization phase and the *g*_Na_ controls mainly the amplitude of the AP [1].

**Fig 6:**
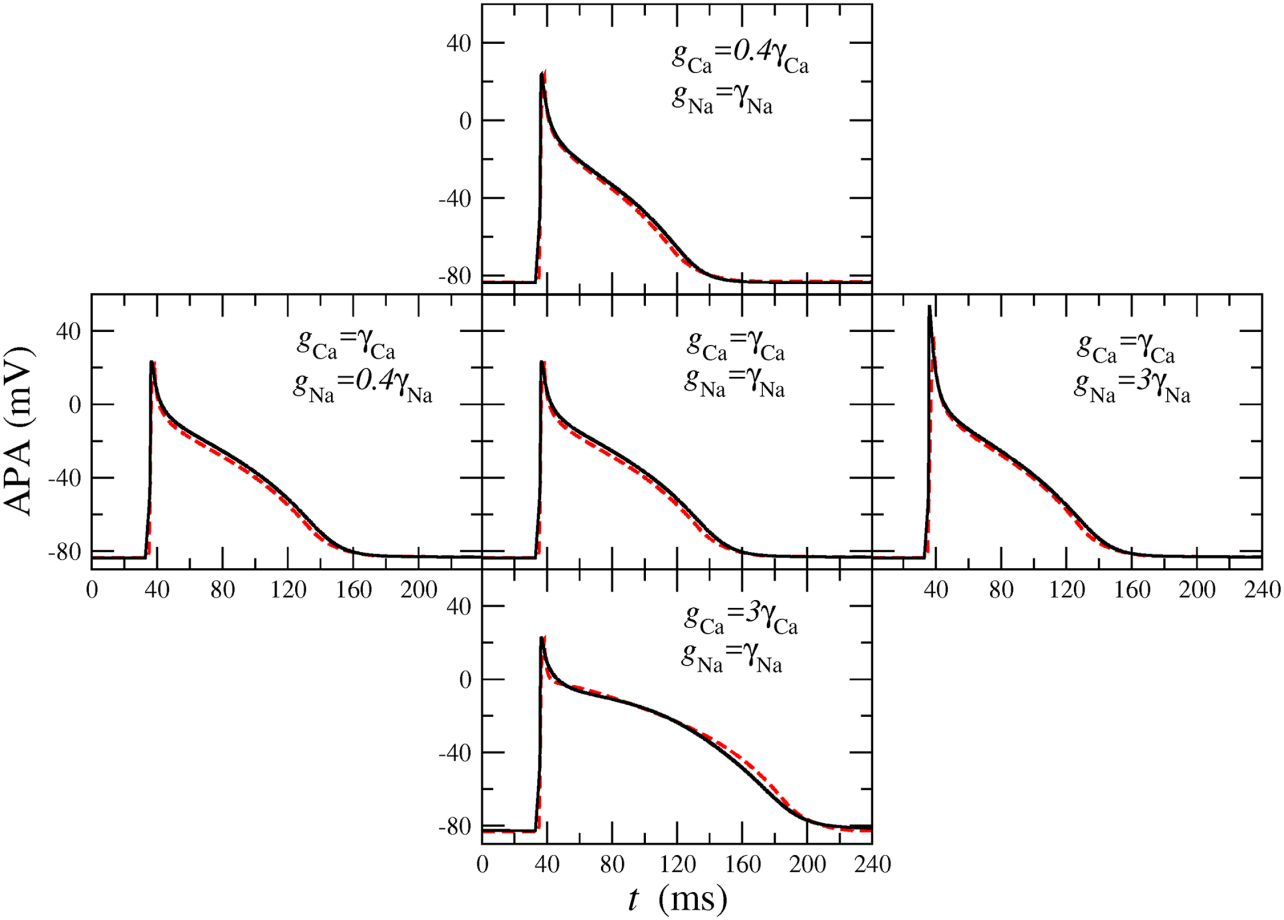
Comparison between CRN model and BOCF model. Five surrogate AP generated with the CRN model (solid lines) for different *g*_Na_ and *g*_Ca_ in comparison with the corresponding AP modeled with the BOCF model (dashed lines). The reference values are the ones corresponding to the remodeling case, namely *γ*_Ca_ = 0.0433 nS/pF and *γ*_Na_ = 7.8 nS/pF.

Fig 6 shows five examples of AP generated with the CRN model, which cover a wide range of APA and APD. In Fig 6 we allow *g*_Na_ and *g*_Ca_ to differ by factors between 40% and 300% from their values *γ*_Na_ = 7.8 nS/pF and *γ*_Ca_ = 0.0433 nS/pF for the electrically remodelled tissue [14]. The corresponding AP modeled with the BOCF model, i. e. for *τ*_fi_ from Eq (4), and *τ*_si_ and *τ*_so1_ obtained from the minimization of $F(\tau_{\text{si}},\tau_{\text{so}1})$ in Eq (6), are shown as dashed lines in the figures. In all cases these reproduce well the AP shapes generated with the CRN model.

To quantify the difference between the AP, we denote by $A_{\text{CRN}}\left(t\right)$ and $A_{\text{BOCF}}\left(t\right)$ their time course, and compute their relative deviation based on the *L*_2_-norm,

$$\begin{array}{c} \Delta A=\frac{\left|\right|A_{\text{BOCF}}\left(t\right)-A_{\text{CRN}}\left(t\right){\left|\right|}_{L_{2}}}{\left|\right|A_{\text{CRN}}\left(t\right){\left|\right|}_{L_{2}}}\;, \end{array}$$ (7)

where

$$\begin{array}{c} {\left||A\left(t\right)|\right|}_{L_{2}}\equiv {\left(\int_{t_{i}}^{t_{f}}A^{2}\left(t\right)dt\right)}^{1/2}\;. \end{array}$$ (8)

The initial time *t*_*i*_ and final time *t*_*f*_ are defined as the times for which *u*(*t*_*i*_) = *u*(*t*_*f*_) = *θ*_0_ with *θ*_0_ = 0.015473 (see S1 Appendix), with opposite signs of the corresponding time derivatives, i.e. $\frac{du}{dt}{|}_{t_{i}}>0$ and $\frac{du}{dt}{|}_{t_{f}}<0$.

**Fig 7:**
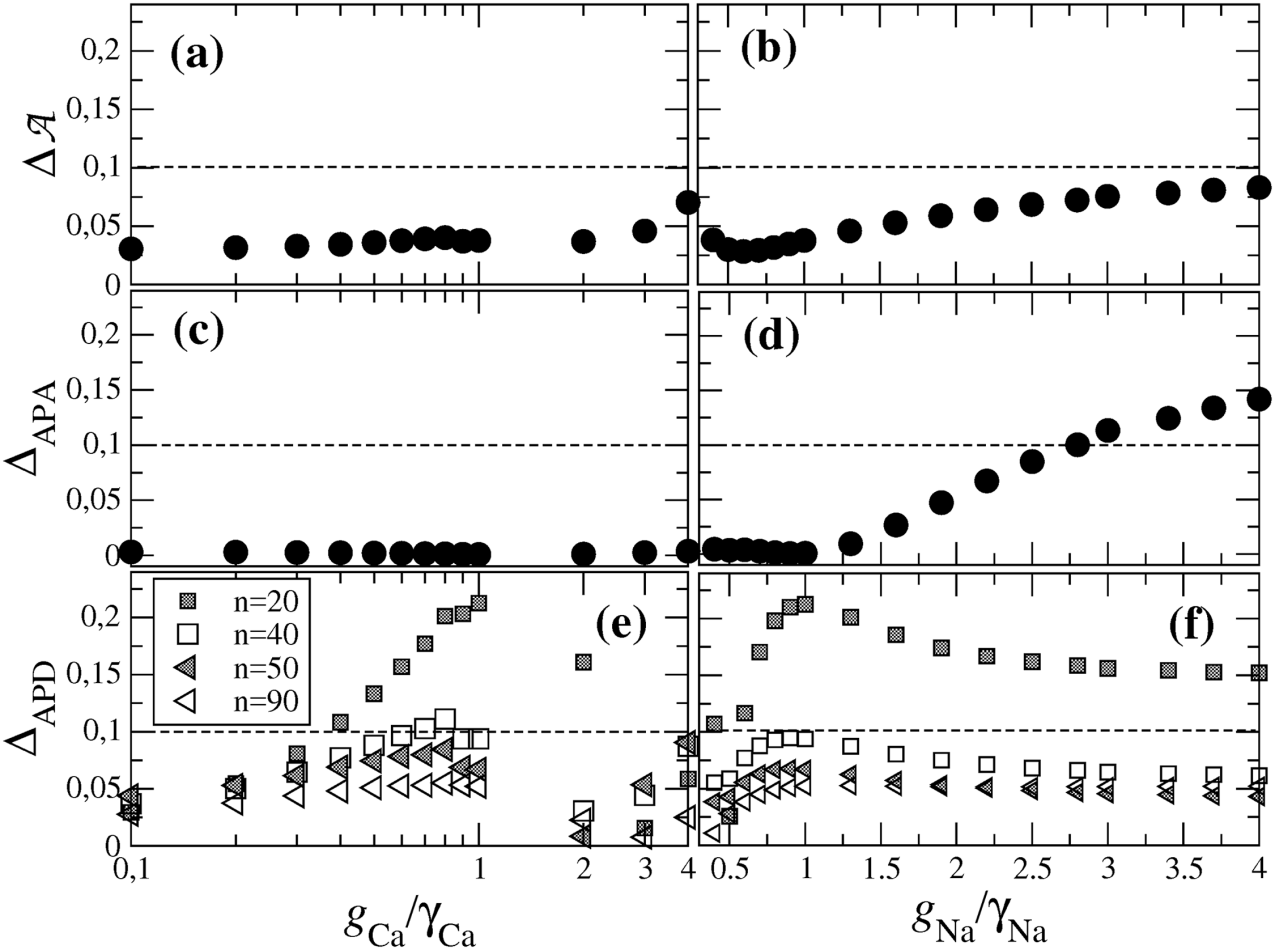
Deviation ΔA (a,b), Δ_APA_ (c,d) and Δ_APD_ (e,f) as a function of *g*_Ca_/*γ*_Ca_ for fixed *g*_Na_ = *γ*_Na_ = 7.8 nS/pF (a,c,e) and as a function of *g*_Na_/*γ*_Na_ for fixed *g*_Ca_ = *γ*_Ca_ = 0.0433 nS/pF (b,d,f). The assignment of the symbols in plot (f) is the same as in plot (e). The dashed line in each plot indicates deviations of 10%.

Fig 7(a) shows that, when keeping *g*_Na_ = *γ*_Na_ fixed, ΔA is below 5% for values of *g*_Ca_ between 10-400% of the reference value *γ*_Ca_. For $g_{\text{Ca}}/\gamma_{\text{Ca}}≳4$, ΔA starts to increase. Likewise, as show in Fig 7(b), ΔA does not exceed 9% when varying *g*_Na_ between 10–400% of *γ*_Na_ for fixed *g*_Ca_ = *γ*_Ca_.

Additionally to the relative deviation between AP, one can compute the relative deviations between the APA and APD_*n*_ retrieved from the BOCF fit,

$$\begin{array}{c} \Delta =\frac{|X_{\text{BOCF}}-X_{\text{CRN}}|}{X_{\text{CRN}}}\;. \end{array}$$ (9)

Here *X* represents either V, giving Δ_APA_ or $D_{n}$, giving Δ_APD_*n*__.

Fig 7(c) and 7(d) show Δ*X*_APA_ as a function of *g*_Ca_/*γ*_Ca_ and *g*_Na_/*γ*_Na_, again for fixed *g*_Na_ = *γ*_Na_ and *g*_Ca_ = *γ*_Ca_, respectively. Corresponding plots of the Δ*X*_APD_*n*__ are shown in Fig 7(e) and 7(f). Fig 7(c) shows that Δ*X*_APA_ is always very small, even for large deviations of *g*_Ca_ from the reference value *γ*_Ca_. By contrast, Δ*X*_APA_ is quite sensitive to variations of *g*_Na_. The deviation becomes larger than 5% for $g_{\text{Na}}/\gamma_{\text{Na}}≳2$.

As for the Δ*X*_APD_*n*__ they are typically below 12% except in the case of APD_20_. The APD_20_ refers to the TMV level closest to the maximum and exhibits larger deviations up to about 20% for even small shape deviations.

All in all, Fig 7 shows that the optimization procedure retrieves acceptable fits of single-cell AP in a wide range of calcium and sodium conductances.

## Robustness with respect to the activation frequency

**Fig 8:**
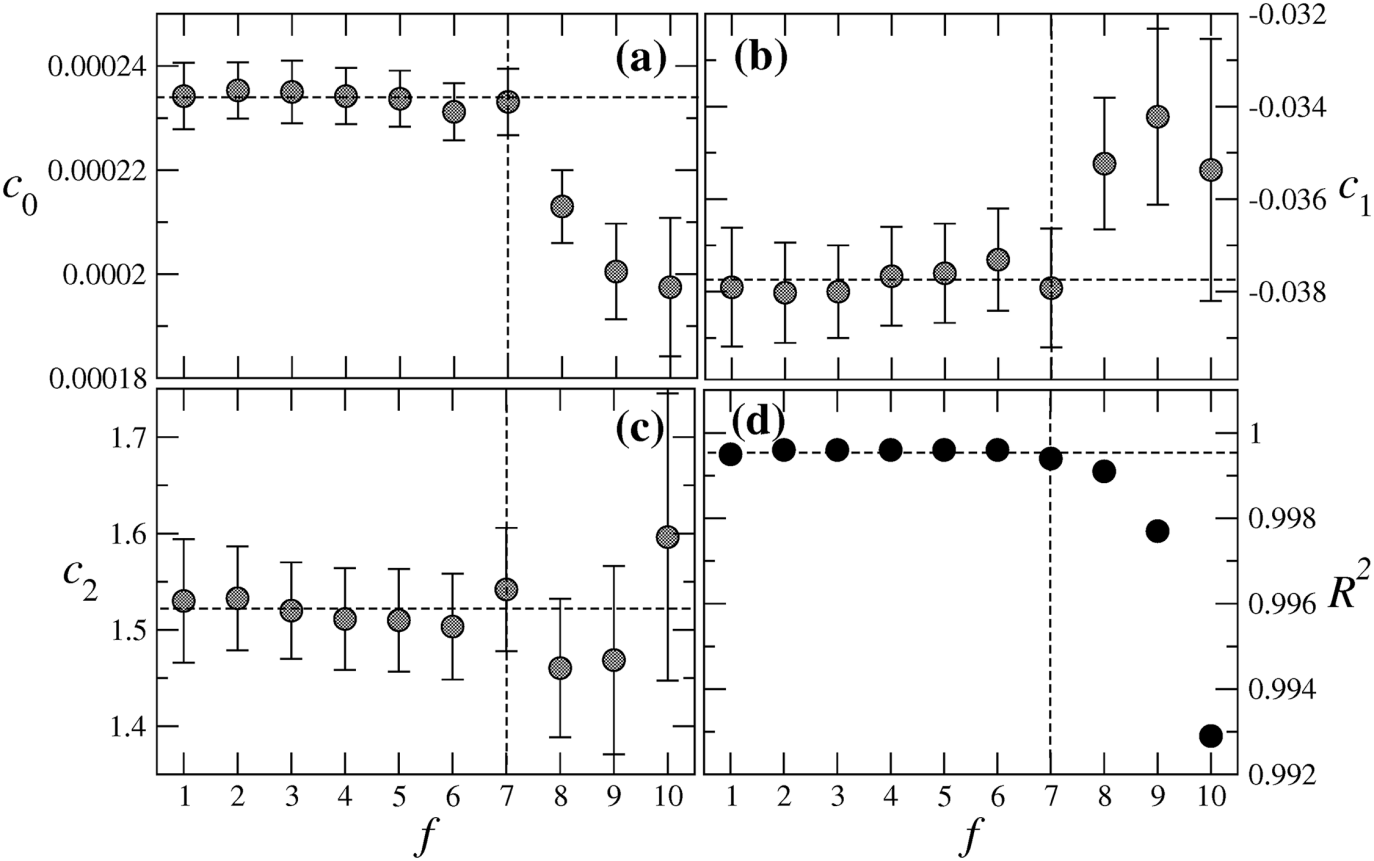
Dependence of the coefficients (a) *C*_0_, (b) *C*_1_, and (c) *C*_2_ in Eq (4) on the activation frequency *f*; (d) the *R*^2^ values of the corresponding fits.

**Fig 9:**
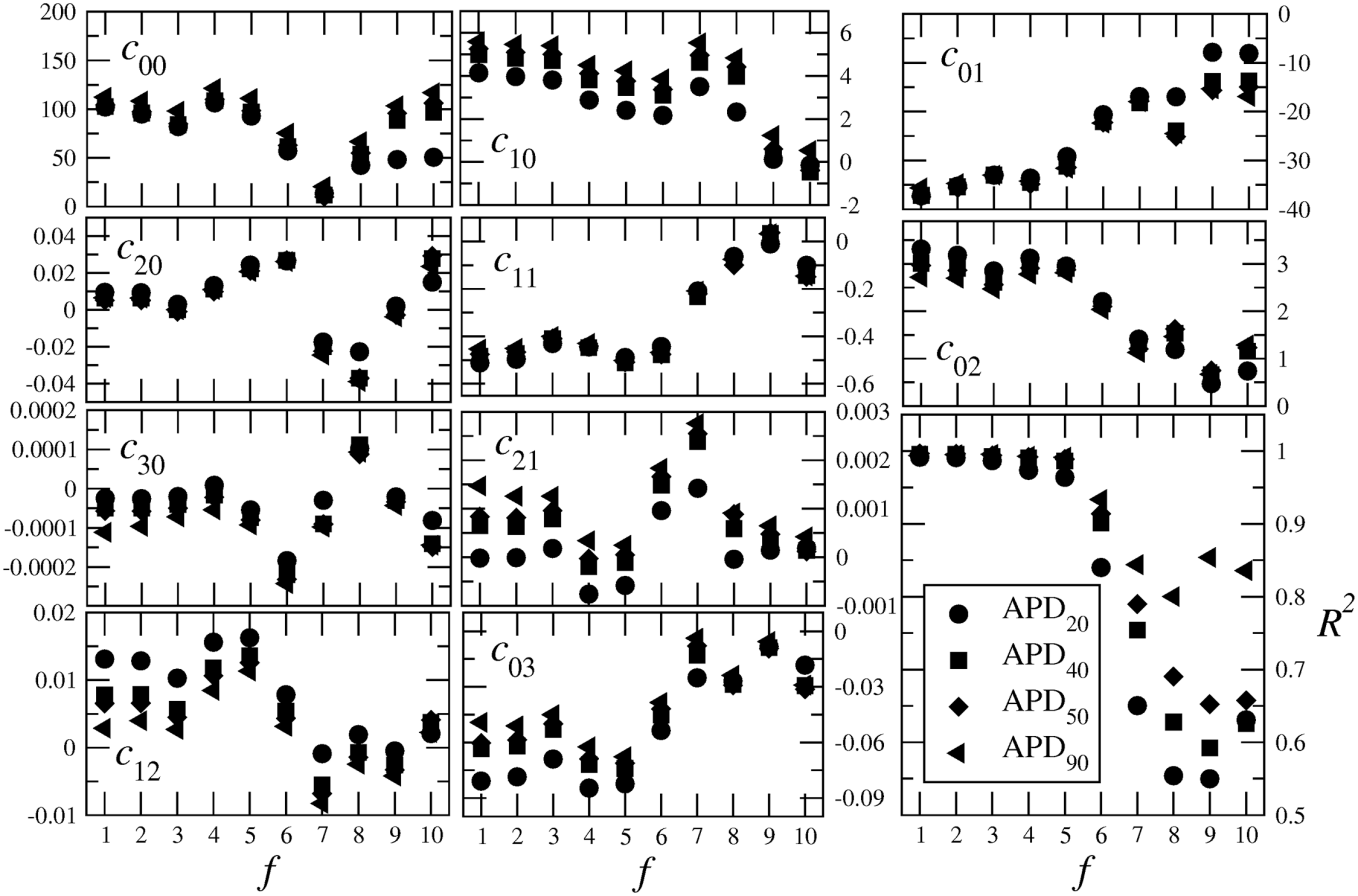
Dependence of the coefficients $c_{mk}^{\left(n\right)}$ in Eq (5) on the activation frequency *f*, where the symbol assignment refers to the different APD_*n*_ as given in the inset of the last graph, which shows the *R*^2^ values of the corresponding fits [Eq (5)].

The optimization procedure described in this paper was illustrated using one single activation frequency, namely *f* = 3 Hz. An important issue is the robustness of the optimization framework for other activation frequencies, which we address in Figs 8 and 9.

Fig 8(a)–8(c) show the variation of the three coefficients in Eq (4) to fit the functional dependence of the APA on the parameter *τ*_fi_. As one sees, all three coefficients are approximately constant for activiations below 7 Hz. In that range of values one also observes a coefficient of determination $R^{2}≳0.999$, as shown in Fig 8(d). For higher frequencies *f*, the coefficients start to vary and the *R*^2^ values of the fits become smaller, indicating the need of higher order polynomials to describe the relation between *τ*_fi_ and APA.

Similar results are obtained for the coefficients used to fit the APD surfaces as functions of the parameters *τ*_si_ and *τ*_so1_. These are shown in Fig 9 and demonstrate that the optmization procedure can be applied faithfully in the range 1 – 6 Hz. Outside this range, polynomials of higher order would be needed for better matches.

**Fig 10:**
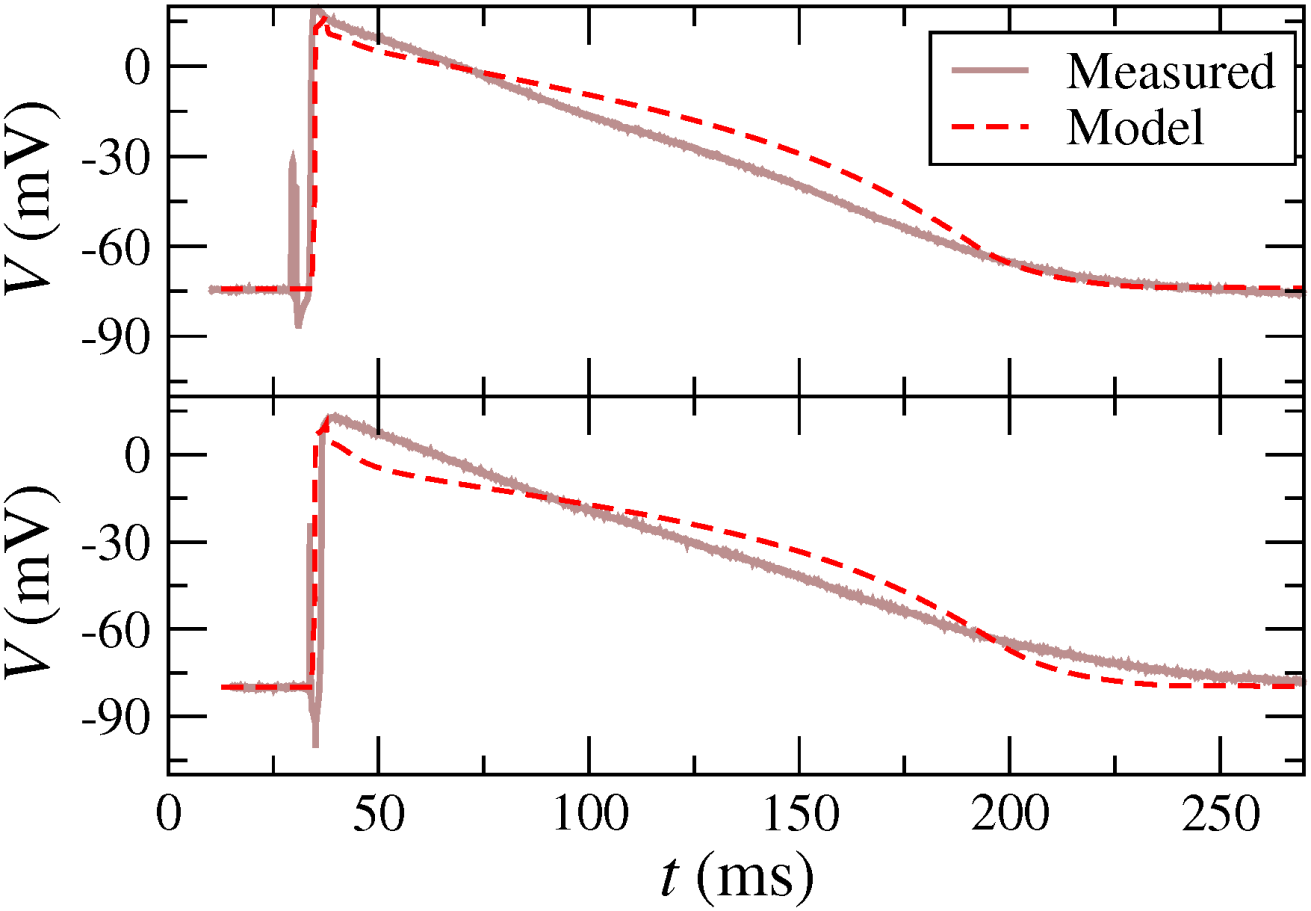
Two illustrative examples of the optimization procedure for fitting AP, taken from sets of measured AP at the University of Dresden.

All in all, this section provides evidence that our optimization procedure derived for an activation frequency of 3 Hz, may also be applicable for frequencies ranging at least between 1 and 6 Hz. Fig 10 shows two illustrative examples of real AP and the respective fit with the optimization procedure. For details about the real data see Ref. [17].

## Conclusions

In this work we showed how to model patient-specific action potentials by adjusting three characteristic time scales, which are associated with the net sodium, calcium and potassium ionic currents. The framework explores the possibilities of parameter adjustment of an atrial physiology model, namely the BOCF model [11], to reproduce AP shapes with a given amplitude, width and duration. The BOCF model is defined through a reaction-diffusion equation, coupled to three equations for gating variables that describe the opening and closing of ionic channels. It is simple enough to guarantee low computational costs for even extensive simulations of spatio-temporal dynamics [18]. Through a semi-analytical approach given in the S1 Appendix we showed why the three ionic currents suffice to derive the main features of empirical AP.

The high flexibility for case-specific applications can be used for clinical purposes. By adjusting a simulation to specific patient conditions one may also analyze numerically the effect of drug therapy under specific conditions. Using the optimization procedure for AP shape adjustment, the three characteristic times are retrieved, which are directly connected to the ion-type specific net currents. AP shapes showing pathological features will be reflected in the values of one (or more) times outside acceptable ranges. Accordingly, one can associate a corresponding net current and therefore identify the class of membrane currents, where pathologies should be present. In this sense the clinical diagnosis can be supported by the modeling. A future application could be to take the retrieved parameters values as a basis for spatially extended simulation by including the diffusion term in Eq (1) [11]. For this, one would need access to conduction properties which then would enable one to model spatio-temporal AP evoluion.

Though our framework is applicable in a quite wide range of values of sodium and calcium conductances, for conductances beyond a few times the reference values for electrically remodelled tissue the matching of AP shapes becomes less accurate. As for changes of the activation frequency *f*, the analysis in the Supporting Information limits the applicability of the AP modeling based on Eqs (4) and (5) to the range *f* = 1 – 6 Hz.

Furthermore, in case information is obtained about AP shapes from different places of the atria, e. g. by using a lasso catheter, a corresponding AP shape modeling would allow one to construct a patient-specific model with spatial heterogeneities. Based on this, it could become possible to generate spatio-temporal activation pattern and to identify possible pathologies associated in the dynamics of the action potential propagation.

## Supporting information

S1 Fig Time evolution of one AP together with each ionic current. (a) AP variable *u* with the stimulus current *J*_stim_, with (b) a close-up for a time interval of 3.5 ms. Vertical dashed lines intersect the AP at one specific dotted line, thus bounding the time intervals corresponding to each region of *u*-values (see text). The ionic currents correspond to (c) the Na channel (*J*_fi_), (d) the Ca channel (*J*_si_), and (e) the K channel (*J*_so_), see S1 Appendix. All currents are given in (ms)^−1^.
(EPS)

S2 Fig Ionic currents as function of the respective gating variables. (a) *J*_fi_ and (b) *J*_si_(*u*, *w*). The red circles indicate the path corresponding to Eqs (1) and (3) and sketched in S1 Fig as a function of time. (c) Dependence of ionic current *J*_so_ on variable *u*.
(EPS)

S3 Fig Time evolution of the four variables of the BOCF model. **(a)** AP variable *u* and the three gating variables **(b)** *v*, **(c)** *w* and **(d)** *s*. The horizontal dotted lines in (a) indicate the ranges of *u*-values, where the evolution of the set of variables changes discontinuously. Vertical dashed lines intersect the AP at one specific dotted line, thus bounding the time intervals corresponding to each region of *u*-values. In several of such time intervals, some of the variables decay exponentially and independently from the other variables, which simplifies the model considerably. In the regions where no exponential evolution is indicated the model follows the reduced system of equations derived in S1 Appendix.
(EPS)

S1 Appendix Dynamical features of the BOCF model: A semi-analytical approach. (TEX)
